# Supplementary material for: SARS-CoV-2 Spike S1-specific IgG kinetic profiles following mRNA or vector-based vaccination in the general Dutch population show distinct kinetics
Source: Sci Rep. 2022 Apr 8;12:5935. doi: 10.1038/s41598-022-10020-6 (PMC8990276; doi:10.1038/s41598-022-10020-6)
Supplement: Supplementary file 1 — Supplementary Information. [file 41598_2022_10020_MOESM1_ESM.pdf]

SARS-CoV-2 Spike S1-specific IgG kinetic profiles following mRNA or vector-based vaccination in the general Dutch population show distinct kinetics

Lotus L. van den Hoogen; Marije K. Verheul; Eric R.A. Vos; Cheyenne C.E. van Hagen; Michiel van Boven; Denise Wong; Alienke J. Wijmenga-Monsuur; Gaby Smits; Marjan Kuijer; Debbie van Rooijen; Marjan Bogaard-van Maurik; Ilse Zutt; Jeffrey van Vliet; Janine Wolf; Fiona R.M. van der Klis; Hester E. de Melker; Robert S. van Binnendijk; Gerco den Hartog

Supplementary Material

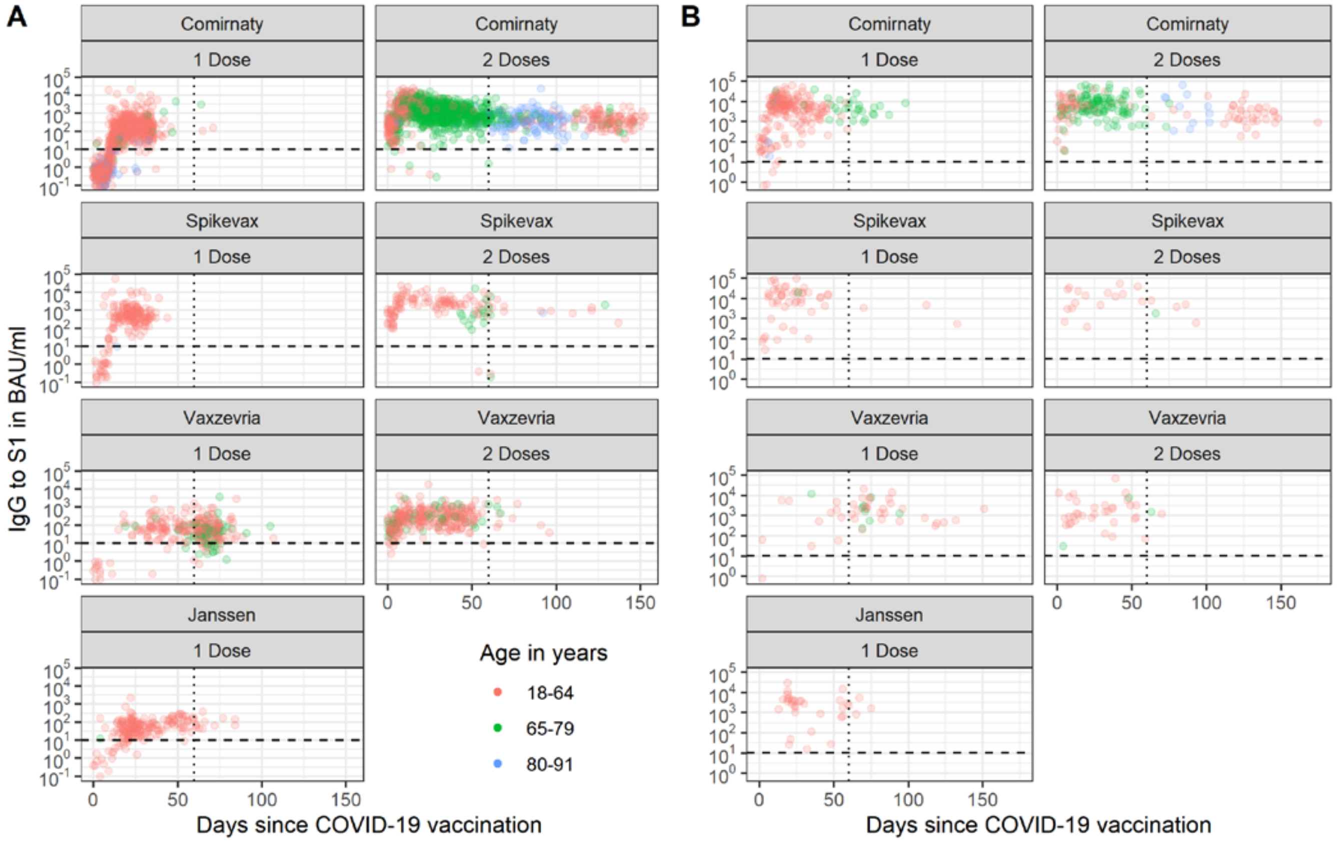

Supplementary Figure 1: Spike S1 immunoglobulin G (IgG) concentrations over time by age group, number of doses and vaccine brand for SARS-CoV-2-naive participants (A) and participants with a SARS-CoV-2 infection history (B). Measurements for a total of 4,110 participants are shown; data left of the dotted vertical line at two months was included in the analyses for adults aged 18-64 years old to enable direct comparison of vaccines (n=2,412). For Comirnaty, data was also analyzed between two and four months following the second dose in SARS-CoV-2-naïve participants across all ages (18-91 years old; n=196). The dashed horizontal line represents the threshold for seropositivity. BAU/mL: binding antibody units per mL; IgG: immunoglobulin G.

Supplementary Table 1: Generalized Additive Model regression results for Spike S1 immunoglobulin G (IgG) concentrations per vaccine type and dose in the SARS-CoV-2-naïve study population aged 18-64 years up to two months following the indicated dose. IgG concentrations in BAU/ml were log10-transformed. All covariates were included in the initial model (time since dose in days, age group, sex and comorbidity) and the final model was created using backward selection by manually dropping the variable with the highest p-value at each step and selecting the more complex model if it resulted in a decrease in Akaike's Information Criterion (AIC) of at least two. No interactions were considered.

	**Comirnaty**		**Spikevax**		**Vaxzevria**		**Janssen**		**Comirnaty**		**Spikevax**		**Vaxzevria**	
**Doses**	**1 Dose**								**2 Doses**					
**N**	791		175		101		186		408		108		265	
	Coef.	p-value	Coef.	p-value	Coef.	p-value	Coef.	p-value	Coef.	p-value	Coef.	p-value	Coef.	p-value
**Age in years**														
18-29	Ref								Ref					
30-44	-0.30	<0.001							-0.28	0.007				
45-64	-0.42	<0.001							-0.30	0.002				
**Sex**														
Male	Ref		Ref				Ref		Ref					
Female	0.15	<0.001	0.23	0.023			0.21	0.006	0.19	0.001				
**Comorbidity**														
None/other*			Ref		Ref								Ref	
High risk**			-0.30	0.019	-0.22	0.045							-0.17	0.005

**Time since dose in days (spline)**		<0.001		<0.001				<0.001		<0.001		<0.001		<0.001

*Other comorbidities: hay fever, skin disease or allergies.

**High risk comorbidities: asthma or other lung disease, cardiovascular disease, diabetes, (history of) cancer, history of transplantation, kidney disease, immune disease, splenectomy, liver disease, rheumatoid arthritis, intestinal disease, neurological disease, or other (open field). Persons with these comorbidities were prioritized during the vaccination campaign in the Netherlands as they were considered high risk for severe COVID-19.

BAU/mL: binding antibody units per mL; IgG: immunoglobulin G; Coef: coefficient; Ref: reference.

Supplementary Table 2: General characteristics of the SARS-CoV-2-naïve study population between two and four months after completion of the Comirnaty vaccination schedule by age category.

	**18 to 64**	**65 to 79**	**80 to 91**
N	35	44	117
Sex			
- Male	11 (31%)	27 (61%)	70 (60%)
- Female	24 (69%)	17 (39%)	47 (40%)
Healthcare worker			
- No	8 (23%)	42 (95%)	117 (100%)
- Yes	27 (77%)	2 (5%)	0 (0%)
Comorbidities			
- Risk group*	15 (43%)	24 (55%)	63 (54%)
- None/other**	20 (57%)	20 (45%)	54 (46%)
Median vaccination interval in days (IQR)	28 (25 – 35)	35 (35 – 36)	35 (35 – 37)
Median time since second dose in days (IQR)	104 (83 – 116)	69 (63 – 80)	86 (75 – 96)

*High risk comorbidities: asthma or other lung disease, cardiovascular disease, diabetes, (history of) cancer, history of transplantation, kidney disease, immune disease, splenectomy, liver disease, rheumatoid arthritis, intestinal disease, neurological disease, or other (open field). Persons with these comorbidities were prioritized during the vaccination campaign in the Netherlands as they were considered high risk for severe COVID-19.

**Other comorbidities: hay fever, skin disease or allergies.

IQR: interquartile range.

Supplementary Table 3: General characteristics of the unvaccinated adults aged 18-64 years up to two months following a positive SARS-CoV-2 test. Participants who reported a positive PCR or antigen test for SARS-CoV-2 up to two months prior to sampling were included.

	**n (%)**
N	114
Sex	
- Male	34 (30%)
- Female	80 (70%)
Age in years	
- 18-29	27 (24%)
- 30-44	37 (32%)
- 45-64	50 (44%)
Healthcare worker	
- No	77 (68%)
- Yes	37 (32%)
Comorbidities	
- Risk group*	28 (25%)
- None/other**	86 (75%)
COVID-19 symptoms when tested	
- No	14 (12%)
- Yes	100 (88%)
Median time since positive SARS-CoV-2 test in days (IQR)	40 (24 – 52)

***** High risk comorbidities: asthma or other lung disease, cardiovascular disease, diabetes, (history of) cancer, history of transplantation, kidney disease, immune disease, splenectomy, liver disease, rheumatoid arthritis, intestinal disease, neurological disease, or other (open field). Persons with these comorbidities were prioritized during the vaccination campaign in the Netherlands as they were considered high risk for severe COVID-19.

**Other comorbidities: hay fever, skin disease or allergies.

IQR: interquartile range, PCR: polymerase chain reaction.
